# Clinical Outcome and Prognostication of Patients With Inflammatory and Immune Myopathies With and Without Chemotherapy in the United States

**DOI:** 10.7759/cureus.96441

**Published:** 2025-11-09

**Authors:** Baljinder Singh, Yixin Chen, Simrandeep K Brar, Elina Zakin

**Affiliations:** 1 Neurology, New York University (NYU Langone Health), New York City, USA; 2 Biostatistics, Virginia Tech University, Blacksburg, USA; 3 Internal Medicine, Texas Tech University Health Sciences Center, El Paso, USA; 4 Neurology, New York University Grossman School of Medicine, New York, USA

**Keywords:** chemotherapy, immune-mediated inflammatory myopathy, inflammatory and immune myopathies, inflammatory myopathy, myopathy, prognostication

## Abstract

Aim

To compare the clinical outcome and in-hospital complication risk with chemotherapy and without chemotherapy in patients with inflammatory and immune myopathies (IIM).

Methods

We obtained data for IIM patients admitted to hospitals in the United States from 2017 to 2018 with a primary diagnosis of IIM using a large national database. We determined the rate and pattern of utilization of associated in-hospital outcomes of chemotherapy in this patient population.

Results

A total of 5,360 patients had IIM, of which 315 (5.8%) received chemotherapy for the underlying malignancies and 5,045 (94.2%) did not. No significant racial or gender discrepancies were observed between the two groups. Patients who received chemotherapy had more in-hospital complications, including sepsis [79 (25.1%) vs. 585 (11.6%), p<0.01] and acute kidney injury [75 (23.8%) vs. 665 (13.2%), p<0.01]. The number of deaths was higher in the chemotherapy group [125 (39.7%) vs. 190 (3.8%)]. Further analysis of patients who died in the chemotherapy group showed that myocardial infarction [20 (6.3%)] and pneumonia [45 (14.3%)] were the leading causes of death.

Conclusion

In hospitalization complications such as sepsis and acute kidney injury were higher in patients who received chemotherapy in IIM patients; however, patients who died had a higher risk of myocardial infarction and pneumonia, so routine cardiac and pulmonary evaluation should be done during hospitalization of this patient population.

## Introduction

Inflammatory and immune myopathies (IIM) encompass a spectrum of conditions, each usually characterized by specific autoantibodies. This includes dermatomyositis, antisynthetase syndrome, immune-mediated necrotizing myositis, inclusion body myositis, overlap syndrome, and polymyositis [1].

IIM is a chronic autoimmune disorder that involves multiple systems in the body, often presenting with myalgias, skin changes, and interstitial lung disease [2]. A study conducted in Norway suggests that approximately one in four individuals diagnosed with IIM develop cancer within three years of the onset of symptoms [3]. Within these categories, distinctions are made based on the presence of specific antibodies and typical clinical phenotypes. Traditionally, inflammatory and immune myopathies (IIM) have been classified based on clinical presentation, electrodiagnostic testing, and muscle biopsy. However, with recent advancements, there has been a significant shift towards classifying IIM based on autoantibodies. This new approach has greatly enhanced our understanding of the various subtypes of IIM, which is critical for evaluation, management, and prognostication [4].

Certain subtypes in these diverse groups of autoimmune spectrum are linked to certain HLA types, supporting a genetic association in specific subsets of IIM. The major histocompatibility complex (MHC) is widely recognized for its substantial genetic contribution to numerous autoimmune diseases. Research focusing on candidate genes has identified a correlation between HLA-DRB10301 and HLA-DQA10501, and the occurrence of IIM in both adult and juvenile Caucasian populations. These HLA associations are more commonly found in patients with anti-Jo-1 or anti-PM-Scl antibodies, indicating a heightened risk for these individuals [5-6]. Additionally, some subtypes of inflammatory and immune myopathies are associated with underlying cancer, while others are not [7]. We have investigated the clinical outcomes and prognostication of patients who were admitted to the hospital with a primary diagnosis of inflammatory and immune myopathy who underwent chemotherapy for their underlying malignancy. Given the limited data on this subject, our study focused on in-hospital complications and explored the primary causes of mortality in these patients.

This article was previously presented at the 2024 Annual Meeting of the American Academy of Neurology in Denver, Colorado, and at the 2024 Annual Meeting of the American Association of Neuromuscular and Electrodiagnostic Medicine in Savannah, Georgia.

## Materials and methods

Our analysis utilized data sourced from the Nationwide Inpatient Sample (NIS) spanning the years 2017 to 2018 [8]. The NIS, a cornerstone of the Healthcare Cost and Utilization Project (HCUP) administered by the Agency for Healthcare Research and Quality (AHRQ), stands as the largest comprehensive inpatient care database in the United States [9]. To offer a national perspective, the NIS draws from a carefully stratified 20% sample of hospitals across the country, representing a substantial volume of approximately seven million hospital stays derived from around 4000 hospitals. This extensive dataset encompasses both clinical and nonclinical variables pertinent to hospital stays, including primary and secondary diagnoses, procedures, admission, and discharge statuses, as well as crucial patient demographic information such as sex, age, race/ethnicity, expected payment source, total charges, and length of stay. To ensure accuracy and reliability, the NIS employs sophisticated hospital and discharge weights, enabling the generation of precise national estimates. Further information on the NIS design and methodology, including comprehensive details, can be accessed on the HCUP website [8].

We used the International Classification of Diseases, tenth revision, Clinical Modification (ICD-10-CM) primary diagnosis codes G72.4, G72.41, and G72.49 to identify the patients admitted with an inflammatory and immune myopathy. Patients who received chemotherapy for underlying malignancies were recognized with ICD-10 codes Z92.6 and Z51.11.

In-hospital complications and comorbid information were obtained from the AHRQ comorbidity data collected for each patient. We used ICD-10-CM secondary diagnosis codes to identify the comorbidities of hypertension (I10), diabetes mellitus (E10, E11), systemic lupus erythematosus (M32.9), Rheumatoid arthritis (M06.9), and Alcohol dependence (F10.2). The ICD-10-CM secondary diagnosis codes were used to identify patients with IIM associated with hospital complications, which were as follows: Pneumonia (J18.9), deep venous thrombosis (I82.40), acute kidney failure (N17), sepsis (A41.9), and myocardial infarction (I21.0). We used ICD-10-CM procedure codes to estimate the percentage of IIM patients who underwent gastrostomy tube (Z93.1) [8].

We also assessed the distribution of patients across teaching vs. non-teaching hospitals. Within the NIS database, information regarding a hospital's location and teaching status was sourced from the American Hospital Association Annual Survey of Hospitals. Teaching hospitals were identified based on the presence of a participating AMA-approved residency program or holding membership in the Council of Teaching Hospitals. Bed sizes of hospitals were further categorized into three subgroups within the NIS database, delineated as small, medium, or large, contingent upon the hospital's location and teaching status. The following categories were defined as In rural areas - Small (1-49 beds), Medium (50-99 beds), and Large (100+ beds); in urban (non-teaching) areas-Small (1-99 beds), Medium (100-199 beds), and Large (200+ beds); In urban (teaching) areas: Small (1-299 beds), Medium (300-499 beds), and Large (500+ beds). The severity of illness was determined utilizing the all patient refined-diagnosis related group (APR-DRG) levels. This assessment incorporated various data elements, including principal and secondary diagnoses coded in the ICD-10-CM, age, sex, and discharge disposition. Severity was stratified into minor, moderate, major, and extreme categories based on the patient's clinical condition. The primary endpoint of this study was mortality at discharge (i.e., dead or alive).

Statistical analysis 

We conducted a comparative analysis of patient demographics, clinical features, in-hospital complications, and outcomes among individuals who were admitted with inflammatory and immune myopathies stratified by their history of chemotherapy use for their underlying malignancies. Our statistical analyses were conducted using weighted numbers to account for the complex sampling design of the NIS database, in accordance with HCUP guidelines. To ensure accuracy and reliability, raw counts obtained from the NIS database were transformed into weighted counts using Statistical Analysis System 9.4 software (SAS Institute Inc., Cary, North Carolina). These weighted counts were then utilized to generate national estimates for our study.

Within the NIS dataset, discharge weight data is denoted as discharge-level weight to discharges in the universe (DISCWT). To ensure national representation, we applied DISCWT to adjust the sampled discharges in the NIS to reflect discharges from all non-rehabilitation, non-long-term acute care community hospitals in the United States.

The discharge sample weights were calculated within each sampling stratum, computed as the ratio of overall discharges to those captured in the NIS. This estimation of overall discharges is derived from a combination of data obtained from hospitals not included in the NIS (sourced from the American Hospital Association) and data from hospitals within the NIS (based on HCUP data). Detailed insights into the estimation process of nationwide hospital discharges can be accessed through the HCUP website. Our analyses were thoroughly conducted, considering the complex sampling design and sample discharge weights of the NIS, in line with HCUP-NIS recommendations. For statistical testing, we employed the Rao-Scott chi-square test for categorical variables to obtain national estimates. These analyses aimed to identify significant differences among patients admitted with inflammatory and immune myopathies who received chemotherapy during or before hospitalization. 

We adjusted for diabetes and alcohol intake because of their prognostic importance in determining the outcome in patients with inflammatory and immune-mediated myopathies. Diabetes and alcohol intake can increase the risk of cardiomyopathy, aspiration pneumonia, sepsis, and myopathy [10-11].

## Results

The primary findings of the study reveal that patients with inflammatory and immune myopathy who underwent chemotherapy experienced higher incidences of acute kidney injury and sepsis during their hospital stay (Table 1). Upon further examination of the chemotherapy group, dividing patients into those who survived and those who did not, we found that the occurrence of myocardial infarction was 16 times more prevalent in the deceased group. Although there was no notable disparity in the occurrence of myocardial infarction between the chemotherapy and non-chemotherapy groups, it emerged as the primary cause of mortality among patients who received chemotherapy. Notably, in-hospital pneumonia was also more prevalent in the deceased patients (Table 2). In general, patients with IIM in the chemotherapy group had a higher mortality (39.6%) in comparison to the non-chemotherapy group (3.1%) (Table 3). Additionally, while the occurrence of acute kidney injury and sepsis was significantly elevated in the chemotherapy group, their rates were not significantly higher in the deceased subgroup, presumably not resulting in the patient’s ultimate demise. The rate of mortality among both groups was not influenced by the size of the hospital or whether the institution was a teaching or non-teaching hospital. Notably, the patients who received chemotherapy for their underlying malignancies had more severe health co-morbidities compared to patients who had no history of chemotherapy use. However, our study has shown that myocardial infarction and pneumonia are the leading causes of death in patients who received chemotherapy. This finding indicates the necessity for formal recommendations specifically tailored to this patient population. 

**Table 1:** Baseline Characteristics, In-Hospital Complications, and Outcomes of Patients With and Without History of Chemotherapy. Chi-square test was used for categorical variables to obtain national estimates. SLE: systemic lupus erythematosus.

Characteristic	Patients with chemotherapy history N=315 (5.8%)	Patients without chemotherapy history N=5045 (94.2%)	p-value	Statistical test
Demographic characteristics				
Women	115 (36.5%)	2200 (43.6%)	0.2606	Chi-square
Race/ethnicity				
White	235 (74.6%)	3352 (66.4%)	0.1631	Chi-square
Black	30 (9.5%)	930 (18.4%)	0.0599	Chi-square
Hispanic	20 (6.3%)	320 (6.3%)	0.9984	Chi-square
Others	30 (9.5%)	445 (8.8%)	0.8440	Chi-square
Mean age ± SD, years	72.7 ± 1.3	67.5 ± 0.5	<0.01	t-test
Comorbid conditions (ICD-10 Code)				
Hypertension (I10)	100 (31.8%)	2270 (45.0%)	<0.05	Chi-square
Diabetes mellitus (E10, E11)	70 (22.2%)	1505 (29.8%)	0.1947	Chi-square
SLE (M32.9)	5 (1.6%)	130 (2.6%)	0.6278	Chi-square
Rheumatoid arthritis (M06.9)	5 (1.6%)	165 (3.3%)	0.4608	Chi-square
Alcohol dependence (F10.2)	1 (0.3%)	70 (1.4%)	1.0	Chi-square
In-hospital complications (ICD-10 Code)				
Pneumonia (J18.9)	45 (14.3%)	420 (8.3%)	0.103	Chi-square
Deep venous thrombosis (I82)	10 (3.2%)	190 (3.8%)	0.9340	Chi-square
Acute kidney failure (N17)	75 (23.8%)	665 (13.2%)	<0.05	Chi-square
Sepsis (A41.9)	79 (25.1%)	585 (11.6%)	<0.005	Chi-square
Pulmonary embolism (I26.9, I26)	1 (0.3%)	95 (1.9%)	0.6217	Chi-square
Myocardial infarction (I21, I22)	20 (6.3%)	370 (7.3%)	0.7713	Chi-square
In-hospital procedures (ICD-10 Code)				
Gastrostomy (Z93.1)	25 (7.9%)	240 (4.8%)	0.2651	Chi-square
Hospital bed size				
Small	65 (20.6%)	925 (18.3%)	0.6421	Chi-square
Medium	55 (17.5%)	1385 (27.5%)	0.0797	Chi-square
Large	195 (61.9%)	2735 (54.2%)	0.2326	Chi-square
Teaching status of admitting hospital				
Non-teaching	45 (14.3%)	815 (16.2%)	0.6987	Chi-square
Teaching	260 (82.5%)	3845 (76.2%)	0.2547	Chi-square

**Table 2:** Baseline Characteristics, In-Hospital Complications, and Outcomes of Alive vs Dead Patients. Chi square test was used for categorical variables to obtain national estimates. SLE: systemic lupus erythematosus.

Characteristic	Alive (N=190)	Dead (N=125)	p-value	Statistical test
Demographic characteristics				
Women	65 (34.2%)	50 (40.0%)	0.5409	Chi-square
Race/ethnicity				
White	145 (76.3%)	90 (72.0%)	0.6117	Chi-square
Black	15 (7.9%)	15 (12.0%)	0.4756	Chi-square
Hispanic	10 (5.3%)	10 (8.0%)	0.4369	Chi-square
Others	20 (10.5%)	10 (8.0%)	0.7238	Chi-square
Mean age ± SD, years	73.97 ± 1.30	70.8 ± 2.21	0.3038	t-test
Comorbid conditions				
Hypertension	55 (28.9%)	45 (36.0%)	0.4713	Chi-square
Diabetes mellitus	55 (28.9%)	15 (12.0%)	<0.005	Chi-square
SLE	5 (2.6%)	0 (0.01%)	1.0	Chi-square
Rheumatoid arthritis	0 (0.01%)	5 (4.0%)	0.3968	Chi-square
In-hospital complications				
Pneumonia	15 (7.9%)	30 (24.0%)	<0.05	Chi-square
Deep venous thrombosis	0 (0.01%)	10 (8.0%)	0.1536	Chi-square
Acute kidney failure	45 (23.7%)	30 (24.0%)	0.9726	Chi-square
Sepsis	50 (26.3%)	30 (24.0%)	0.7955	Chi-square
Myocardial infarction	0 (0.01%)	20 (16.0%)	<0.05	Chi-square

**Table 3:** Multivariate Analysis Evaluating the Effect of Chemotherapy on In-Hospital Mortality in Patients with Inflammatory and Immune Myopathies (NIS 2017-2018). The database was adjusted for the history of alcohol intake and diabetes. OR: odds ratio, CI: confidence interval.

	Patients without history of chemotherapy N = 5045 (94.2%)	Patients with history of chemotherapy N= 315 (5.8%)	Unadjusted		Adjusted	
Outcomes			OR (95% CI)	p value	OR (95% CI)	p value
Alive at discharge	4885 (96.8)	190 (60.4%)	1.0 (Reference)		1.0 (Reference)	
In hospital death	160 (3.1%)	125 (39.6%)	20.066 (10.87 to 37.05)	<0.001	19.748(10.74 to 36.31)	<0.001

## Discussion

Certain aspects of the analysis have direct implications for interpretation. We utilized the NIS database, which does not provide information on the specific types of chemotherapeutic agents used, the particular subtypes of immune-mediated myositis, or their association with specific malignancies. We employed ICD-10 codes to identify cases of inflammatory and immune myopathies. While the exact accuracy of these codes is not fully established, hospitals do utilize these ICD codes for Medicare reimbursement, ensuring diligent verification for billing claims. Additionally, ICD codes have a precedent of being used in clinical research, supporting their validity for use in our study [12].

Systemic manifestations of inflammatory and immune myopathies (IIM) extend beyond skin findings to include pulmonary and gastrointestinal findings [13]. While the association between subsets of IIM and various malignancies is well-established [14]. However, to our knowledge, this is the first study to examine in-hospital complications and primary causes of death among patients with IIM who received chemotherapy. Considering our findings of the substantial risk of myocardial infarction in this patient population when hospitalized, a comprehensive cardiac evaluation should be conducted upon admission, including an electrocardiography (EKG), cardiac enzyme levels, and an echocardiogram, even in the absence of acute myocardial infarction. Various chemotherapies, such as anthracyclins and tyrosine kinase inhibitors, are considered cardiotoxic at higher doses and primarily present with congestive heart failure [15]. As results of our study highlight the heightened risk of myocardial infarction-associated mortality in these patients. The earlier intervention and monitoring can potentially reduce the risk of sudden death in this patient population. We recommend that, in addition to the comprehensive cardiac workup on admission, these patients should also undergo a timely and thorough cardiac evaluation following hospital discharge. 

Although the incidence of acute kidney injury (AKI) and sepsis was higher in the chemotherapy group, the difference between the deceased and surviving patients was not statistically significant. This does not negate the need for measures to prevent AKI and sepsis in these patients. We recommend a multidisciplinary approach for admitted patients, involving oncology, rheumatology, and neurology consultants on admission. Additionally, the potential for tumor lysis syndrome associated with cancer and chemotherapy can elevate the risk of AKI [16]. Given that IIM patients are often treated with immunosuppressive medications, which can further increase the risk of infection and sepsis, safety precautions should be taken to prevent hospital-acquired infections.

## Conclusions

Our results suggest that patients with inflammatory and immune-mediated myopathies. tend to have a higher rate of mortality when admitted to the hospital, with myocardial infarction being the leading cause of death among these patients. This finding highlights the necessity for strategies that ensure comprehensive cardiac evaluation both upon admission and throughout the course of hospitalization. Implementing such measures could potentially improve outcomes and reduce mortality in this vulnerable patient population.
